# Association Between rs217727 and rs2839698 H19 Polymorphisms and Obesity

**DOI:** 10.1007/s10528-023-10418-5

**Published:** 2023-06-16

**Authors:** Soudeh Ghafouri-Fard, Maryam Dadyar, Shahryar Azizi, Solat Eslami, Bashdarm Mahmud Hussen, Mohammad Taheri, Fariborz Rashnoo

**Affiliations:** 1 Department of Medical Genetics, Shahid Beheshti University of Medical Sciences, Tehran, Iran; 2 Phytochemistry Research Center, Shahid Beheshti University of Medical Sciences, Tehran, Iran; 3 Department of Surgery, Erfan Niayesh Hospital, Tehran, Iran; 4 Dietary Supplements and Probiotic Research Center, Alborz University of Medical Sciences, Karaj, Iran; 5 Department of Medical Biotechnology, School of Medicine, Alborz University of Medical Sciences, Karaj, Iran; 6 Department of Clinical Analysis, College of Pharmacy, Hawler Medical University, Kurdistan Region, Erbil, Iraq; 7 Institute of Human Genetics, Jena University Hospital, Jena, Germany; 8 Urology and Nephrology Research Center, Shahid Beheshti University of Medical Sciences Tehran, Tehran, Iran; 9 Skull Base Research Center, Loghamn Hakim Hospital, Shahid Beheshti University of Medical Sciences, Tehran, Iran

**Keywords:** H19, lncRNA, Polymorphism, Obesity

## Abstract

Obesity is a worldwide health problem with an increasing trend. This condition has a significant genetic background. H19 lncRNA has been shown to protect from dietary obesity through decreasing levels of monoallelic genes in brown fat. In the current study, we aimed to find the association between two possibly functional H19 polymorphisms, namely rs217727 and rs2839698 and obesity in Iranian population. These polymorphisms have been shown to affect risk of some obesity-related conditions in different populations. The study included 414 obese cases and 392 controls. Notably, both rs2839698 and rs217727 were associated with obesity in the allelic model as well as all supposed inheritance models. In addition, after adjustment for gender, all *P* values remained significant. For rs2839698, the OR (95% CI) for T allele vs. C allele was 3.29 (2.67–4.05) (*P*-value < 0.0001). In the co-dominant model, both TT and CT genotypes were found to confer risk of obesity compared with CC genotype (OR (95% CI)= 14.02 (8.39–23.43) and 9.45 (6.36–14.04), respectively). Similarly, combination of TT and CT genotypes had an OR (95% CI) = 10.32 (7.03–15.17) when compared with CC genotype. For rs217727, the T allele was found to exert a protective effect (OR (95% CI) = 0.6 (0.48–0.75)). Moreover, in the co-dominant model, OR (95% CI) values for TT and TC genotypes vs. CC genotype were 0.23 (0.11–0.46) and 0.65 (0.49–0.87), respectively. Taken together, H19 polymorphisms may affect risk of obesity in Iranian population. It is necessary to conduct functional studies to confirm a causal relationship between the rs217727 and rs2839698 polymorphisms and obesity.

## Introduction

Obesity is a worldwide health problem with an increasing trend over the past decades (Abarca-Gómez et al. 2017). This trait has both polygenic and early-onset monogenic forms (Loos and Yeo 2022). Gene discovery investigations have shown common genetic and biological underpinnings for both forms (Loos and Yeo 2022). Based on twin, family and adoption studies, the heritability index for obesity is estimated to be 40–70% (Maes et al. 1997; Elks et al. 2012). Gene discovery approaches for polygenic obesity include candidate gene and genome-wide linkage studies (Loos and Yeo 2022). In the current study, we used the latter approach to find the association between two H19 polymorphisms, namely rs217727 and rs2839698 and obesity in Iranian population. H19 is a long non-coding RNA (lncRNA) that is merely transcribed from maternally inherited alleles (Leighton et al. 1995). The gene coding this lncRNA is located near the *insulin-like growth factor 2* (*IGF2*) gene. H19 lncRNA has been shown to protect from dietary obesity through decreasing levels of monoallelic genes in brown fat (Schmidt et al. 2018). Another study has indicated that enhancement of function of H19 would have anti-obesity impacts (Li et al. 2021). Moreover, expression assays have shown down-regulation of H19 in subcutaneous adipose tissues of obese females, compared to normal-weight controls (Daneshmoghadam et al. 2021). Expression levels of H19 have been inversely correlated with obesity indices and homeostasis model assessment of insulin resistance values. Therefore, H19 has been found to be involved in the obesity-associated conditions (Daneshmoghadam et al. 2021).

Association between single nucleotide polymorphisms (SNPs) within H19 and human disorders have been verified in several studies (Ghapanchi et al. 2020; Lu et al. 2016; Harati-Sadegh et al. 2018). Particularly, rs217727 polymorphisms in the exon 5 of this gene has been shown to affect cancer susceptibility (Wang et al. 2019). Most notably, H19 rs217727 polymorphism has been associated with susceptibility to type 2 diabetes in Iranian population (Ghaedi et al. 2018) and ischemic stroke in Chinese population (Zhu et al. 2018).rs2839698 is another SNP within H19 whose association with cancer susceptibility has been assessed in recent years (Yu et al. 2020; Safari et al. 2019). Notably, *in vitro* studies have confirmed the functionality of this polymorphism (Cao et al. 2020). Based on the results of luciferase reporter assay, rs2839698 variant affects the binding of miRNAs to H19 (Cao et al. 2020). Besides, individuals having CT/TT genotypes of rs2839698 have been found to express higher levels of H19 compared with those carrying the CC genotype (Cao et al. 2020).

Based on the above-mentioned evidence on the possible role of H19 in the obesity, we aimed to assess the association between rs217727 and rs2839698 polymorphisms of this lncRNA and obesity in Iranian population to find clues about genetic background of this trait in this population.

## Materials and Methods

### Cases and Controls

The study included 414 obese cases and 392 controls. Cases were selected from patients referred for sleeve gastrectomy in Erfan Niayesh Hospital, Tehran, Iran, during 2021–2022. They had either BMI ≥ 40 kg/m^2^ without coexisting medical problems or BMI ≥ 35 kg/m^2^ with 1 or more severe obesity-associated problems (Mancini 2014). Control subjects were healthy persons with BMI ≤ 25. Informed consent forms were signed by all cases and controls. The study protocol was approved by ethical committee of Shahid Beheshti University of Medical Sciences (Ethical code: IR.SBMU.RETECH.REC.1402.147).

### Genotyping

Genomic DNA was extracted from blood samples of cases and controls using the modified salting out method as described by Nasiri et al. (2005). rs217727 and rs2839698 genotypes were determined using the protocol described previously (Safari et al. 2019). Tetra primer-ARMS-PCR technique was used for genotyping. Primers for genotyping rs2839698 were as follow: Forward inner primer (C allele): CTGATGTCAGTGAGGAGTGTGGAGTATGC, Reverse inner primer (T allele): GCCCTGTCTACACGATGCCTGGACA, Forward outer primer: 5′-GAAAAAGACCTGGCTAGGACCGAGGAG and Reverse outer primer: ATCAAACCCTGCCCACCAGCTCCCCTC. Products sizes for C and T alleles were 187 and 273 bp, respectively. Two outer primers produced an amplicon with the size of 404 bp.

Primers for genotyping rs217727 were as follow: Forward inner primer (T allele): ACATCTTCATCGCCACCCCCTGCTGT, Reverse inner primer (C allele): TGTGATGGCTGGTGGTCAACCGTTCG, Forward outer primer: GACTAAGGAATCGGCTCTGGAAGGTGAG and Reverse outer primer: GATGGAGGAAACAGAGTCGTGGAGGCTT. Sizes of amplicons for T and C alleles were 205 and 248 bp, respectively. Outer primers produced an amplicon with size of 399 bp.

**Fig. 1:**
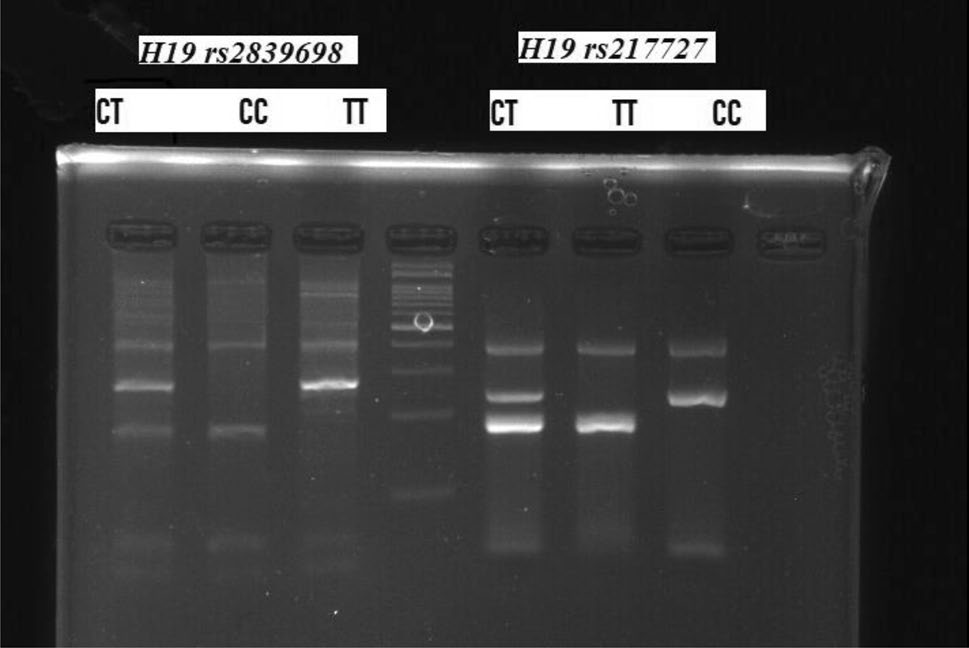
Size of amplicons for rs217727 and rs2839698 genotypes as shown on 2% agarose gel

PCR conditions were as follow: denaturation step at 95 °C for 5 min, 30 cycles of denaturation at 95 °C for 30 s, annealing (at 59 °C for rs2839698 and 69 °C for rs217727) for 45 s and extension at 72 °C for 45 s. The final extension time at 72 °C for 5 min. Figure 1 shows the size of amplicons for each genotype of these SNPs.

### Statistical Methods

SPSS v.22.0 (SPSS Inc., Chicago, IL) and SNP Analyzer 2.0 were used for statistical assessments. Allele and genotype frequencies of rs217727 and rs2839698 variants were compared between study subgroups using the chi-square test. Relative risks (odds ratios) for effect alleles and genotypes were calculated by logistic regression. Adjusted relative risks were calculated with gender as covariate. Associations between genomic variants and obesity risk were assessed in co-dominant, dominant, recessive and over-dominant models. The results of association analysis were described as Odds ratios (OR) and 95% confidence interval of OR (95% CI), *P*-value and FDR adjusted *q*-values. The FDR adjusted *q*-values were calculated by analyzing a stack of p values in column analyses by GraphPad Prism version 9.0. *P* values less than 0.05 were considered as statistically significant. Accordance of genotype distributions with Hardy–Weinberg equilibrium, haplotypes and linkage disequilibrium (LD) blocks were assessed using SNP Analyzer 2.0.

Association of obesity risk with haplotypes was investigated using a haplotype-specific test with one degree-of-freedom. D′ and *r* parameters were calculated for assessment of linkage between rs217727 and rs2839698 variants.

Graphics were created using GraphPad Prism version 9.0 for Windows, GraphPad Software, La Jolla California USA.

## Results

General information about cases and controls is summarized in Table 1. Age of cases and controls tended to be matched (*P*-value = 0.0498).

**Table 1:** Demographic data of cases and controls

Parameters	Cases	Controls
Male, *n*	54	99
Female, *n*	360	293
Age, mean ± SD (y)	37.42 ± 22.16	40.58 ± 19.34
BMI	Mean ± SD: 41.13±5.88	Range: 18.5–24.9

**Fig. 2:**
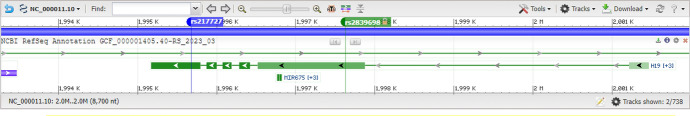
Locations of rs217727 and rs2839698 in the *H19* gene. The rs217727 is located in Exon 6 at position 586670, and the rs2839698 is located in the Exon 2, at position 5213 of the H19 gene on chromosome 11. The H19 gene is located on minus strand and the variant alleles for rs217727 are C>T and for rs2839698 are C>T, G

Position of rs217727 and rs2839698 in relation with *H19* gene is shown in Fig. 2.

The allele and genotypes distribution of both SNPs (rs2839698 and rs217727) was significantly different in the obese patients from that in the normal BMI controls. The allele distribution was also calculated at gender level between obese patients and normal controls by chi-square test. There was also a significant difference between male and female obese patients compared with relevant normal controls (Table 2).

**Table 2:** Genotype and allele frequencies of two *H19* SNPs in study groups by gender (*n*, %)

	Genotypes/Alleles of rs2839698									Genotypes/Alleles of rs217727								
	C/C	C/T	T/T	χ^2^	*P*	C	T	χ^2^	*P*	C/C	C/T	T/T	χ^2^	*P*	C	T	χ^2^	*P*
Normal controls																		
Males (*n* = 99)	50 (50.5)	35 (35.4)	14 (14.1)			135 (68.2)	63 (31.8)			37 (37.4)	52 (52.5)	10 (10.1)			126 (63.6)	72 (36.4)		
Females (*n* = 293)	153 (52.5)	118 (40.3)	22 (7.5)			424 (72.4)	162 (27.6)			144 (49.1)	124 (42.3)	25 (8.5)			412 (70.3)	174 (29.7)		
Total (*n* = 392)	203 (51.8)	153 (39)	36 (9.2)			559 (71.3)	225 (28.7)			181 (46.2)	176 (44.9)	35 (8.9)			538 (68.9)	246 (31.4)		
Obese group																		
Males (*n* = 54)	4 (7.4)	34 (63)	16 (29.6)	28.5	<0.0001	42 (38.9)	66 (61.1)	24.6	<0.0001	34 (63)	18 (33.3)	2 (3.7)	9.56	0.008	86 (79.6)	22 (20.4)	10.6	0.001
Females (*n* = 360)	35 (9.7)	244 (67.8)	81 (22.5)	146	<0.0001	314 (43.6)	406 (56.4)	108	<0.0001	212 (58.9)	139 (38.6)	9 (2.5)	14.6	0.001	563 (78.2)	157 (21.8)	8.4	0.004
Total (*n* = 414)	39 (9.4)	278 (67.1)	97 (23.4)	175	<0.0001	356 (43)	472 (57)	132	<0.0001	246 (59.4)	157 (37.9)	11 (2.7)	22.9	<0.0001	649 (78.4)	179 (21.6)	19.7	<0.0001

According to the SNP database, the wildtype alleles for both rs2839698 and rs217727 are C on minus strand. The T alleles were the minor allele for both SNPs and considered as effect alleles

C/C, homozygous reference; C/T, heterozygous and T/T, homozygous mutant for rs2839698 SNP (based on SNP database)

C/C, homozygous reference; C/T, heterozygous and T/T, homozygous mutant for rs217727 SNPs (based on SNP database)

Genotype frequencies are shown in parentheses. χ^2^ test and *P* value are presented for obese groups vs. normal BMI control subjects

**Fig. 3:**
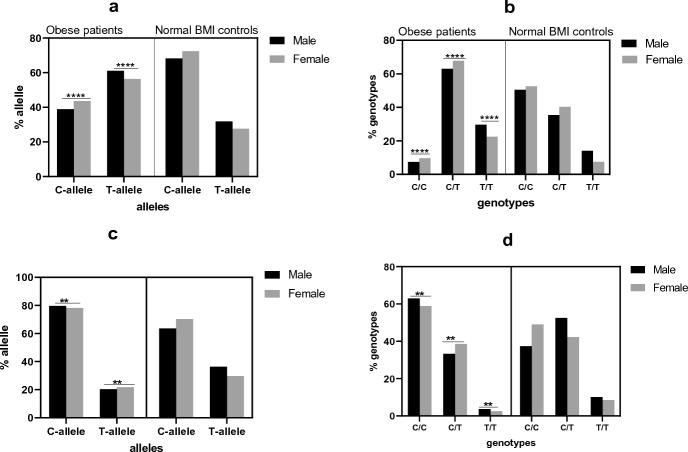
Allele and genotype distribution of rs2839698 (**a**, **b**) and rs217727 (**c**, **d**) variants in the *H19* gene among obese patients and normal BMI controls and their subgroups at gender level. Significant differences in genotype and allele distribution in the study subgroups vs. respective normal controls are shown with stars

The results for allele and genotype distribution are shown in Table 2 and Fig. 3. Gender-based analyses also confirmed that distribution of alleles and genotypes of rs2839698 and rs217727 is different between each subgroup of patients compared with matched controls.

Distribution of rs2839698 and rs217727 genotypes in the control group but not in the cases was in accordance with Hardy-Weinberg equilibrium (Table 3).

**Table 3:** The results of exact test for Hardy-Weinberg equilibrium (*P* values and genotype distributions are shown)

	rs2839698			Hardy-Weinberg *P*-value	rs217727			Hardy-Weinberg *P*-value
	CC	CT	TT		CC	CT	TT	
Obese group	97	278	39	**<0.00001**	246	157	11	**0.01**
Normal BMI controls	203	153	36	0.35	181	176	35	0.39

*P* values less than 0.05 consider as significance

Notably, both rs2839698 and rs217727 were associated with obesity in the allelic model as well as all supposed inheritance models (Table 4). In addition, after adjustment for gender, all P values remained significant.

**Table 4:** Association between rs2839698 and rs217727 variants and obesity in different models (allelic and genotypes)

rsID	Models	OR (95% CI) (1)	*P*-value (1)	FDR q-Value (1)	OR (95% CI) (2)	*P*-value (2)	FDR *q*-value (2)
rs2839698	Allele model T vs. C	3.29 (2.67–4.05)	<0.0001	<0.0001	3.38 (2.74–4.17)	<0.0001	<0.0001
	Co-dominant TT vs. CC CT vs. CC	14.02 (8.39–23.43)	<0.0001	<0.0001	15.74 (9.18–26.99)	<0.0001	<0.0001
		9.45 (6.36–14.04)	<0.0001	<0.0001	9.4 (6.31–14.01)	<0.0001	<0.0001
	Dominant TT+CT vs. CC	10.32 (7.03–15.17)	<0.0001	<0.0001	10.46 (7.1–15.43)	<0.0001	<0.0001
	Recessive TT vs. CT+CC	3.02 (2–4.56)	<0.0001	<0.0001	3.26 (2.14–4.97)	<0.0001	<0.0001
	Over dominant TT+CC vs. CT	0.31 (0.23–0.41)	<0.0001	<0.0001	0.32 (0.24–0.43)	<0.0001	<0.0001
rs217727	Allele model T vs. C	0.6 (0.48–0.75)	<0.0001	<0.0001	0.61 (0.49–0.77)	0.00002	0.00002
	Co-dominant TT vs. CC CT vs. CC	0.23 (0.11–0.46)	<0.0001	<0.0001	0.23 (0.11–0.48)	0.00007	0.00003
		0.65 (0.49–0.87)	0.0042	0.0008	0.67 (0.5–0.9)	0.007	0.001
	Dominant TT+CT vs. CC	0.58 (0.44–0.77)	0.0002	0.00006	0.6 (0.45–0.79)	0.0004	0.0001
	Recessive TT vs. CT+CC	0.27 (0.13–0.55)	0.0003	0.00007	0.28 (0.14–0.57)	0.0004	0.0001
	Over dominant TT+CC vs. CT	1.33 (1–1.76)	0.047	0.008	1.3 (0.98–1.73)	0.06	0.01

Unadjusted Odds Ratios (plus Confidence Intervals) and adjusted Odds Ratios by gender are reported for effect alleles and genotypes

For rs2839698, the OR (95% CI) for T allele vs. C allele was 3.29 (2.67–4.05) (*P*-value < 0.0001). In the co-dominant model, both TT and CT genotypes were found to confer risk of obesity compared with CC genotype (OR (95% CI) = 14.02 (8.39–23.43) and 9.45 (6.36–14.04), respectively). Similarly, combination of TT and CT genotypes had an OR (95% CI) = 10.32 (7.03–15.17) when compared with CC genotype.

For rs217727, the T allele was found to exert a protective effect (OR (95% CI) = 0.6 (0.48–0.75)). Moreover, in the co-dominant model, OR (95% CI) values for TT and CT genotypes vs. CC genotype were 0.23 (0.11–0.46) and 0.65 (0.49–0.87), respectively.

**Fig. 4:**
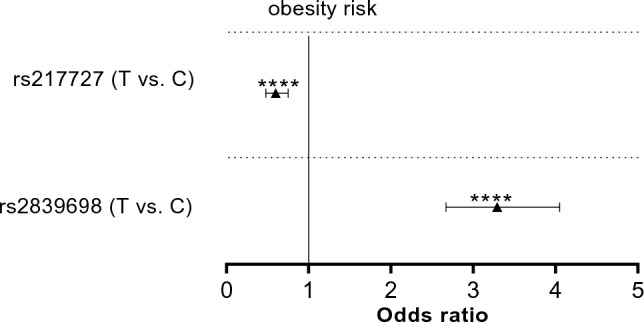
The results of risk association for rs2839698 and rs217727 alleles. Data on the right of Y axis indicates causative effects toward the risk and the data on the left indicates protective effects. The effective alleles (T) were tested against C alleles. The Odds Ratios (plus Confidence Intervals) are reported on the *X* axis in a linear scale (**** *p* < 0.0001)

Figure 4 shows the results of association studies in allelic model. The effective T allele of rs217727 showed a significant protective effect against the risk for obesity and the effective T allele of rs2839698 showed a significant causative effect toward the risk for obesity.

In the co-dominant model, the effective TT and CT genotypes of rs2839698 vs. CC genotype showed a significant protective effect against the risk for obese group. The effective TT and CT genotypes of rs217727 vs. CC genotype showed a significant causative effect toward the risk for obese group. The effective genotypes TT+CT vs. CC in dominant model, TT vs. CT+CC in recessive model for rs217727 and the effective genotype TT+CC vs. CT for rs2839698 in over-dominant model showed a significant protective effect against the risk for obesity. However, the effective genotype TT+CT vs. CC in dominant model and TT vs. CT+CC in recessive model for rs2839698 and the effective genotype TT+CT vs. CT for rs217727 in over-dominant model showed likely significant causative effects toward the obesity risk.

Based on the calculated D and r values, the assessed two polymorphisms within *H19* gene were in moderate linkage disequilibrium (LD) in total study population. These two polymorphisms demonstrated *D*′ = 0.23, *r*^2^ = 0.015 (*P* value < 0.05).

Notably, CT and TC haplotypes (corresponding to rs2839698 and rs217727, respectively) were found to be protective haplotypes against obesity. On the other hand, TT and CC haplotypes were risk haplotypes. Table 5 shows the results of haplotype analyses.

**Table 5:** The results of haplotype analyses for rs2839698 and rs217727 variants

rs2839698	rs217727	Case	Control	Freq.	OR (95% CI)	*P*-value	Adjusted *P*-value
C	C	0.34	0.46	0.39	2.89 (2.34–3.57)	<0.0001	<0.0001
T	C	0.44	0.22	0.34	0.43 (0.34–0.55)	<0.0001	<0.0001
C	T	0.089	0.25	0.17	0.52 (0.42–0.64)	<0.0001	<0.0001
T	T	0.12	0.06	0.08	2.44 (1.5–4.01)	0.0002	0.0002

**Fig. 5:**
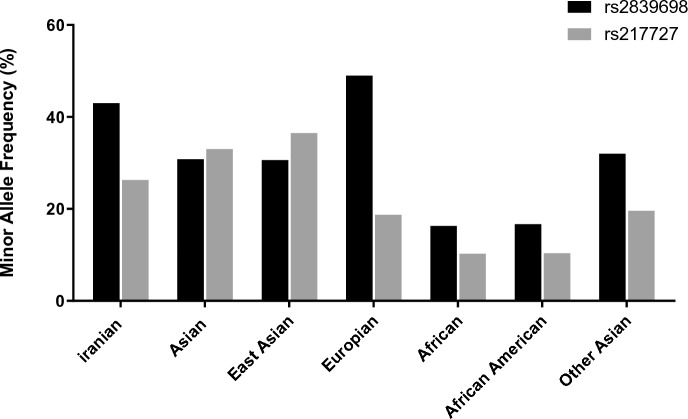
Minor allele frequencies for two H19 SNPs in Iranian samples compared with ALFA project populations

Figure 5 and Table 6 show the frequencies of mentioned polymorphisms in different populations.

**Table 6:** The results of frequencies for two H19 SNPs alleles in Iranian samples compared with ALFA project populations

Population	Sample size (allele No.)		Ref Allele (frequency, %)		Alt Allele (frequency, %)	
	rs2839698	rs217727	rs2839698 (C Allele)	rs217727 (C Allele)	rs2839698 (T Allele)	rs217727 (T Allele)
Iranian	1612	1612	0.567	0.736	0.433	0.264
Asian	386	3778	0.692	0.668	0.308	0.332
East Asian	314	3028	0.694	0.634	0.306	0.366
European	28732	266138	0.502	0.812	0.498	0.188
African	2656	5646	0.836	0.898	0.164	0.102
African American	2582	5434	0.832	0.896	0.168	0.104
Other Asian	72	750	0.68	0.8	0.32	0.2

## Discussion

Identification of genetic *loci* that affect risk of obesity can lead to early detection of at risk persons and management of obesity-related comorbidities. Recent studies have shown the importance of lncRNAs in adipocyte lipid metabolism and related disorders (Zhang et al. 2021). Based on their diverse regulatory roles, lncRNAs have been suggested as promising targets for treatment of obesity and related metabolic disorders (Zhang et al. 2021). Moreover, lncRNAs can influence susceptibility to adipocyte dysfunction-induced diseases, particularly insulin resistance and diabetic complications (Zhang et al. 2021). The current study aimed at investigation of two functional polymorphisms within H19 lncRNA and risk of obesity in Iranian population. As expected, both rs2839698 and rs217727 were associated with obesity in the allelic model as well as all supposed inheritance models. Moreover, haplotype analyses confirmed the observed associations.

H19 can affect adipocyte differentiation of bone marrow mesenchymal stem cells via epigenetic regulation of histone deacetylases (Huang et al. 2016). Moreover, overexpression of H19 has been shown to promote adipogenesis and mitochondrial respiration in brown adipose tissue through recruitment of PEG-inactivating H19-MBD1 complexes (Schmidt et al. 2018). This lncRNA has a fundamental role in the regulation of thermogenic gene program and metabolic pathways in this tissue (Schmidt et al. 2018).

Based on the results of luciferase reporter assay, rs2839698 variant affects the binding of miRNAs to H19 (Cao et al. 2020). T allele rs2839698 have been found to be associated with higher levels of H19 expression (Cao et al. 2020). In the current study, T allele of this SNP was found to confer risk of obesity which is in line with the role of H19 in obesity and the effect of T allele of this SNP on expression of H19. Notably, T allele of this SNP has also been regarded as a protective allele against colorectal cancer in Chinese individuals, particularly among some subclasses of these individuals such as overweight individuals (BMI ≥ 24) (Yu et al. 2020).

Moreover, H19 is a risk locus for some obesity-related disorders such as diabetes (Ghaedi et al. 2018), ischemic stroke (Zhu et al. 2018) and coronary artery disease (Zhang et al. 2017). For rs217727, the T allele was found to exert a protective effect. While a recent study in Iranian population has shown association between this allele and risk of type 2 diabetes (Ghaedi et al. 2018), this allele has been shown to be a risk allele for preeclampsia in this population {Harati-Sadegh, 2018 #1040}. There are several lines of evidence linking obesity, type 2 diabetes and preeclampsia. In fact, insulin resistance that leads to pre-pregnancy obesity or too much weight gain during gestation is linked with a decrease in cytotrophoblast migration and uterine spiral artery remodeling, which in turn induce a series of events leading to preeclampsia {Lopez-Jaramillo, 2018 #1041}. The casual effects of H19 polymorphisms on these events should be assessed in future studies.

Finally, we compared allele frequencies of mentioned polymorphisms in different populations. As expected, allele frequencies reported in Iranian population were close to reports from Asian and European regions, but different from Africans.

Thus, genetic polymorphisms within this lncRNAs are appropriate targets for association studies in the field of obesity. However, the impact of mentioned H19 polymorphisms in the regulation of H19 roles in this tissue has not been investigated. The current study provided clues for association between two H19 polymorphisms and obesity in Iranian population. The mechanism underlying these associations should be investigated in functional studies. Besides, the association between expression of H19 and mentioned variants should be assessed in obese individuals as well as controls.

## Data Availability

All data generated or analyzed during this study are included in this published article [and its supplementary information files].
